# The slow violence of racism on Asian Americans during the COVID-19 pandemic

**DOI:** 10.3389/fpubh.2022.958999

**Published:** 2022-10-26

**Authors:** Gloria Wong-Padoongpatt, Aldo Barrita, Anthony King, Michelle Strong

**Affiliations:** Psychological and Brain Sciences, Department of Psychology, University of Nevada, Las Vegas, NV, United States

**Keywords:** anti-Asian racism, everyday racism, well-being, COVID-19, Asian Americans

## Abstract

Racism against people of Asian descent increased by over 300% after the COVID-19 pandemic outbreak in the United States, with one in five Asian Americans reporting direct experiences with overt discrimination. Large-scale efforts and resources initially, and quite understandably, prioritized investigating the physiological impact of the coronavirus, which has partially delayed research studies targeting the psychological effects of the pandemic. Currently, two studies tracked the unique relationships between psychosocial factors, such as experiencing everyday racism, and the self-reported wellbeing of Asian Americans in the United States and compared these associations with Latinx Americans. Study 1 (April 2020–April 2021) examined how Asian and Latinx Americans varied in their levels of wellbeing, fear of the coronavirus, internalized racism, and everyday experiences with racism. Study 2 (September 2021–April 2022) included the same variables with additional assessments for victimization distress. We used the *CDC Museum COVID-19 Timeline* to pair collected data from our studies with specific moments in the pandemic—from its known origins to springtime 2022. Results highlighted how slow and deleterious forms of racist violence could wear and tear at the wellbeing of targeted people of color. Overall, this research underscores the possible hidden harms associated with slow-moving forms of racism, as well as some of the unseen stressors experienced by people of color living in the United States.

## Introduction

In the United States, the COVID-19 pandemic served as a catalyst for the perpetuation of violence toward people of color (1–3). These examples included individuals acting on long-standing fears about people of Asian descent, particularly newer immigrants to the United States. For ease of nomenclature, we will use the term *Asian American* to include all people of Asian descent residing in the United States. In addition to this, as suggested by the American Psychological Association (APA)'s best practices for bias-free language (4), we will use labels consistent with Asian American or other racial groups, such as Latinx American and White American.

The 1882 Chinese Exclusion Act marked the first significant U.S. law to restrict any form of immigration (5). Decades later, anti-Asian sentiments were further exemplified by the reprehensible treatment of Asian immigrants at Angel Island in 1910 and the targeted detainment of Japanese Americans in U.S. internment camps during World War II (6, 7). Historic events such as these have prompted researchers to broaden the definition of racism-related violence to include microaggressive acts, as well as other unseen or less seen attacks, in an attempt to address and raise awareness about the multi-sided injustices people of color have often been forced to endure throughout the U.S. history (8–10). More recently, hateful sentiments aimed at specific groups and people have spread throughout many cyber platforms, such as Reddit, Twitter, and Facebook, which may elevate the potential for portions of these platforms to become havens for promoting and sustaining racism (11–13).

Since the start of the pandemic, race-related violence and hate crimes against Asian Americans and Asian-looking people have exponentially increased by 339%, compared with an overall 11% increase in other hate crimes (14). Adding to this trend, a recent report by the Center for the Study of Hate and Extremism (15) revealed that one in five Asian Americans reported a direct experience with overt discrimination during the pandemic years. Furthermore, Nguyen et al. (16) analyzed negative sentiments on Twitter, which appeared in 2.3 million tweets during the early months of the COVID-19 pandemic. They found that race-related tweets containing anti-Asian sentiments rose ~55% in a single month alone (i.e., February to March 2020). Another study by Hswen et al. (17) investigated a different aspect of anti-Asian sentiments on Twitter and indicated that the hashtag #ChineseVirus was associated with anti-Asian sentiments 50.4% of the time, compared to 19.7% for the hashtag #Covid19. These results reveal that racist sentiments can appear in a variety of online contexts—even if they were not originally intended for that purpose.

Yet, promisingly, recent efforts to end the blatant attacks and other forms of race-related hate against Asian Americans have grown considerably (2, 3). For example, healthcare leaders have started to recommend the use of non-bias language around the COVID-19 pandemic in an attempt to destigmatize the associations between Asian Americans and the pandemic (12, 17). In other instances, stronger partnerships have started to form between professionals, community leaders, and grassroots organizations as a way to better understand and combat the underlining hate that many Asian American communities are facing, as well as the experiences of people who are misperceived as being of Asian descent (15). By both renewing and strengthening these communal relationships, a core objective is to build effective solutions to potentially halt the continued onslaught of discrimination against Asian Americans.

Taken together, rampant anti-Asian sentiments have become a major health issue for Asian Americans today (2, 18–21). But even before the more recent contributions to this trend, Asian Americans have largely been neglected in and excluded from discussions of U.S. racism; instead, they are more often given the *model minority* stereotype (22), which depicts all Asian people as highly educated and financially stable, compared with other people of color (23). Asian Americans, however, have continuously reported racism-related attacks, both overt and covert (24)—despite arguments that this stereotype reflects a positive image of Asian people and their cultures. Many of the offenses faced by Asian Americans, both past and present, are connected to nativistic and xenophobic racial microaggressions (25), which are considered daily stressors (26) that can significantly impact both psychological (27) and physiological health (28). Due to the continuation of anti-Asian violence in the United States, further investigation of this significant public health concern is critically needed.

Hence, we launched two studies that engaged environmentalist Rob Nixon's slow violence theory (29), along with multicultural psychologist Derald W. Sue's microaggression theory (25), to examine the nuanced impact of the COVID-19 pandemic on Asian Americans compared with that on Latinx Americans. Previous research on Asian and Latinx Americans has reported that these groups tend to have similar experiences with everyday racism, particularly around others perceiving them as perpetual foreigners or less “American” than U.S. residents with a white complexion (21, 30, 31). According to Nixon's slow violence theory (29), violence can occur as either fast and attention-grabbing (e.g., physical attacks in public) or slow and hidden from view (e.g., internalizing racism psychologically). To complement this theoretical framework, Sue et al. (25) scholarship on microaggressions—everyday forms of discrimination—maps on to this slow form of racist violence toward people of color. Empirical research on racial microaggressions has also supported these associations and shown a robust negative effect on the wellbeing of Asian and Latinx Americans (27, 32, 33). Moreover, Wong-Padoongpatt et al. (2) found that Asian Americans were more likely to internalize and believe racist sentiments toward their group than Latinx Americans during the COVID-19 pandemic. These can be understood as insidious types of slow violence of racism that can wear down people of color's sense of self and community.

Past scholarship on racism suggests that these types of beliefs and their associated acts tend to fluctuate with the concurrent sociopolitical events and environments. One study by Barrita et al. (32) found that Asian and Latinx Americans frequently share common discriminatory experiences related to others presuming they may have undocumented immigration statuses. Importantly, findings from that study indicated that Asian Americans reported experiencing more microaggressions around their perceived foreignness during the COVID-19 pandemic than during the times before the pandemic. According to the Racial Position Model (31), the social-racial hierarchy in the United States is shaped across two main dimensions—cultural foreignness and perceived inferiority—to position each racial group. From the perspective of this model, Asian and Latinx Americans, compared with White and Black Americans, are continuously racialized as foreigners and perpetually unable to assimilate to the U.S. culture (31, 32). Asian and Latinx Americans have reported being discriminated based on assumptions of nationality, immigration status, and race (30–32). Therefore, the current studies use Latinx Americans as a comparison community for Asian Americans, given their shared experiences of racialization.

Notably, medical experts have demonstrated that an individual's fear of the coronavirus can potentially amplify the damaging effects of the virus (34–36). Other studies have also shown that fear is often directly associated with the transmission rates of infectious diseases, as well as their rates of mortality and morbidity (34). Yet, most strategies for combating COVID-19 have almost exclusively focused on containing infections, distributing effective vaccinations, and improving general treatment rates (34), without many underlying mechanisms in these associations receiving considerable attention. As more time has passed, however, there has been a growing concern among the general public regarding the psychosocial effects of the COVID-19 pandemic, such as xenophobic racism and other victimization experiences.

Thus, the current two studies tracked the relationships between different psychosocial factors and the wellbeing of Asian and Latinx Americans. Due to the dynamic nature of the pandemic, cross-sectional designs were used to evaluate multiple psychological factors related to racism, fear of the coronavirus, and their associations with one's wellbeing at different time points of the pandemic. This approach allowed for timely data collection without the additional resources needed for a longitudinal design; at the start of this project, it was also unclear how long the pandemic would last. Moreover, we incorporated novel measurements developed specifically for the pandemic, such as COVID-related victimization distress. Study 1 (*N* = 366) explored the effects of internalized racism, everyday racism, and fear of the coronavirus on overall wellbeing during the time frame of the nationwide lockdown and the first vaccine release that occurred. Study 2 (*N* = 185) examined the dynamics between the same psychosocial factors with additional assessments of victimization distress during the months following the widespread availability of COVID-19 vaccines in the United States.

## Study 1

Study 1 (20 April 2020–27 April 2021) examined the relationships between psychosocial factors of everyday racism, internalized racism, and fear of the coronavirus with overall wellbeing among Asian and Latinx Americans. According to the *CDC Museum COVID-19 Timeline* (37), the first case in the United States appeared in mid-January 2020, and the World Health Organization (WHO) declared COVID-19 a global pandemic on 11 March 2020. A few days later, former U.S. President Donald J. Trump declared COVID-19 a nationwide emergency. Despite this declaration, Trump and his administration were simultaneously associating the COVID-19 outbreak with China and the Chinese people by using references, such as “Chinese Virus” and “Kung Flu” in public forums. A recent study by Chong and Chen (11) testified to the far-reach of these words and found Donald Trump was, by far, the most influential promoter of #Chinavirus and #Chinesevirus for the entire Twitter network.

Given this dramatic shift in current anti-Asian sentiments, it was hypothesized that Asian Americans would be more negatively impacted overall during the pandemic with lower overall wellbeing, more exposure to everyday racism, more internalized racism, and more fear of the coronavirus than Latinx Americans. Moreover, it was hypothesized that overall wellbeing would be strongly associated with everyday racism, internalized racism, and fear of the coronavirus for Asian Americans, compared with Latinx Americans, that is, race would moderate the relationships between these psychosocial factors and wellbeing.

## Materials and methods

### Participants

Data for study 1 were collected online during the COVID-19 pandemic, with all participant responses coming from a diverse respondents from Southwest University in the United States. Participants were recruited from an undergraduate psychological participant pool and were compensated course credit for their participation in the study. The final sample in this study consisted of 366 respondents between 18 and 47 years (*M*_age_ = 19.68, *SD* = 2.83) and a gender breakdown of 68% women, 31.1% men, and 0.9% “other.” The overall racial breakdown of the sample was 48.1% Asian American and 51.9% Latinx American. The sample included 71 foreign-born and 284 U.S.-born participants. There were 11 participants identified as international students. A total of six participants were removed for residing outside the United States. See Table 1 for more details of the study's demographic characteristics and descriptive statistics.

**Table 1:** Demographic characteristics and descriptives of study variables compared across Asian and Latinx Americans for study 1 (April 2020–April 2021).

**Variables**	**Race**					
	**Asian Americans**		**Latinx Americans**		**Full sample**	
	***M***	***SD***	***M***	***SD***	***M***	***SD***
Age	19.43	2.11	19.90	3.35	19.68	2.83
SES	5.73_a_	1.46	5.03_b_	1.64	5.36	1.59
	*N*	%	*N*	%	*N*	%
**Gender**
Women	102_a_	58.0	147_b_	77.4	249	68.0
Men	73_a_	41.5	41_b_	21.6	114	31.1
Other	1	0.6	2	1.0	3	0.9
**Generation status**
1^st^	15	8.5	7	3.7	22	6.0
1.5	35_a_	19.9	14_b_	7.4	49	13.4
2^nd^	108_a_	61.4	143_b_	75.3	251	68.6
3^rd^	9	5.1	14	7.4	23	6.3
4^th^ or more	2	1.1	8	4.2	10	2.7
Other	7	4.0	4	2.1	11	3.0
Total	176	48.1	190	51.9	366	100

SES, socioeconomic status. SES was from 1 to 10, with higher scores representing higher perceived SES. Subscript letters indicate statistically significant (p > 0.05) between group differences.

#### Procedures

A university institutional review board reviewed and approved this study to assure compliance with federal and university regulations regarding human participants. Informed consent was obtained from all participants before their participation. Eligibility for the inclusion in the study is given as follows: 18+ years old, U.S. resident during the COVID-19 pandemic, and fluent in the English language. Respondents completed the survey questions online regarding their experiences during the COVID-19 pandemic.

#### Measures

##### Race and other demographic characteristics

Specific racial identities were assessed as part of the demographic questionnaire (Asian Americans and Latinx Americans), along with participants' age, gender, socioeconomic status (SES), and generational status. These demographic questions were delivered at the end of the study to avoid possible priming effects. As previously mentioned, the primary objective of this study was to examine whether there were significant racial differences across different psychosocial factors, particularly related to one's race and the pandemic, for Asian and Latinx Americans during the early stages of the COVID-19 pandemic. Only monoracial people were included in this study since the main study variables were race-related.

##### Wellbeing

The WHO Well-Being Index-5 (WHO-5) (38) consists of five positively worded items that reflect the presence or absence of wellbeing in a person's life (e.g., “Over the last 2 weeks, I have felt cheerful and in good spirits”). Assessment items were rated on a 6-point scale, ranging from 0 (*at no time*) to 5 (*all the time*), with a possible raw score ranging from 0 to 25. The general scoring rule for the WHO-5 is multiplying the raw score by 4 so that the final score range is between 0 (i.e., representing the lowest level of wellbeing) and 100 (i.e., representing the highest level of wellbeing). Topp et al. (39) conducted a systematic review of the WHO-5 and established the two most commonly used cutoff scores: (1) *reduced wellbeing* with a score <50 and (2) *clinical depression* with a score <28. The WHO-5 scale has been validated consistently with diverse samples (39) and has been previously used to assess the wellbeing implications of racist experiences (40, 41). Wang et al. (41) found a strong Cronbach's alpha (α = 0.84) using an exclusive Asian American sample. Cronbach's alpha value of the five items for study 1 were comparable at 0.86 for the Asian American sample, 0.89 for the Latinx American sample, and 0.88 for the final sample (both groups). Study 2 also had high internal reliability with an alpha of 0.89 for the full sample, and 0.88 and 0.89 for the Asian and Latinx American samples, respectively.

##### Everyday racism

The Everyday Discrimination Scale (EDS) (42) consists of nine items presenting possible experiences with discrimination (e.g., “Were you treated with less respect than other people”). The participants reported how often they experience each of the scenarios depicted by using a 4-point scale, ranging from 1 (*never*) to 4 (*often*). Another major focus of this study was to explore the perceived shift in racism-related experiences during the COVID-19 pandemic. Therefore, we modified each EDS item with the words “before the COVID-19 pandemic” and “during the COVID-19 pandemic” to assess perceived experiences with everyday racism during the time frame. Higher scores indicated more consistent experiences with everyday discrimination. Previous studies have shown good internal reliability of the EDS, with α = 0.93 for Latinx American samples (30) and α = 0.86 for Asian American samples (2). In study 1, the scale items for the final sample were comparable at α = 0.86 (assessing events before the pandemic) and α = 0.88 (assessing events during the pandemic). The internal consistency level for the Asian American sample was α = 0.86 before the pandemic and α = 0.90 during the pandemic. For the Latinx American sample, the level was α = 0.87 before the pandemic and α = 0.87 during the pandemic. Study 2 had high internal reliability for the full sample, with an alpha level of 0.88 before the pandemic and 0.88 during the pandemic. For Asian and Latinx American samples, the alpha value ranged from 0.86 to 0.90.

##### Internalized racism

The Emotional Responses subscale within the Appropriated Racial Oppression Scale (ER-AROS) (43) is a 7-item subscale that measures emotional reactions people of color might experience in relation to their own racial identity or racial group to indicate whether an individual is potentially internalizing their experiences with racism. The participants were asked to rate their level of agreement for each scale item, using anchors from 1 (*strongly disagree*) to 7 (*strongly agree*). Higher scores suggest greater levels of internalized racism. The ER-AROS showed high internal reliability with samples of Latinx and Asian Americans, with α = 0.83 (43). Similar levels have also been found for Asian and Latinx American samples in studies specific to the pandemic, α = 0.79 and α = 0.82, respectively (2). In study 1, the ER-AROS demonstrated high Cronbach's alpha for all participants: Asian Americans (α = 0.82), Latinx Americans (α = 0.79), and final sample (α = 0.87). Study 2 also showed a high-reliability alpha of 0.84 for the full sample, with Asian and Latinx samples, 0.85 and 0.81, respectively.

##### Fear of coronavirus

The Fear of COVID-19 Scale (FCV-19S) (34) consists of seven statements denoting behaviors or situations that describe a fear of the COVID-19 virus (e.g., “My hands become clammy when I think about coronavirus-19”). The participants reported their level of agreement for each statement using a Likert-type scale that ranges from 1 (*strongly disagree*) to 5 (*strongly agree*). The scores were summed to reflect an overall score ranging from 7 to 35. Higher scores indicated a greater fear of the coronavirus. The FCV-19S showed high internal reliability for Latinx Americans, with α = 0.87 (44), and Asian Americans, with α = 0.89 (45). Study 1 showed comparable results, with an alpha of 0.87 for all participant groupings. Study 2 also had high internal reliability, with an alpha of 0.87 for the full sample, 0.85 and 0.88 for Asian and Latinx American samples, respectively.

#### Statistical analyses

Partial correlation analysis along with analysis of variance (ANOVA) of the demographic characteristics and study variables was used to examine differences among the sample while holding everyday experiences with racism before the pandemic constant (see Table 2). Comparison analyses were performed for certain demographic characteristics as they related to race and wellbeing, which determined the covariates used in subsequent analyses. The findings determined that gender, generational status, and SES would be held constant within the main study analyses.

**Table 2:** Correlations and ANOVA results for demographics and study variables for study 1 controlling for everyday racism before the pandemic.

**Variable**	**Everyday racism**	**Internalized racism**	**COVID fear**	**Well-being**
**Partial correlations** ***r***
Age	0.17*	0.04	0.08	−0.06
SES	0.01	−0.10	0.07	0.28***
Generation status	−0.07	−0.13*	0.05	0.04
**ANOVA** ***F***
Gender	0.04	5.60${}_{\text{m}}^{*}$	17.94${}_{\text{w}}^{***}$	5.75${}_{\text{m}}^{*}$
Race	7.99${}_{\text{a}}^{**}$	24.07${}_{\text{a}}^{***}$	1.80	1.61

SES, socioeconomic status. SES was from 1 to 10, with higher scores representing higher perceived SES. Generation Status: 1 = 1st generation, 2 = 1.5 generation, 3 = 2nd generation, 4 = 3rd generation, 5 = 4th and higher generation. Gender: subscript m denotes higher mean for men, subscript w denotes higher mean for women. Race: subscript a denotes higher mean for Asian Americans.

^*^p < 0.05,

^**^p < 0.01,

^***^p < 0.001.

Analysis of covariance (ANCOVA) was conducted on the main outcome and moderating variables—wellbeing, everyday racism, internalized racism, and fear of COVID-19—while controlling for a participants' gender, generational status, and SES. Perceived experiences of everyday racism before the COVID-19 pandemic were also controlled for to better assess the shift in participant experiences during the pandemic.

SPSS PROCESS Macro (46) was used to determine whether one's race moderated the relationship between psychosocial factors and overall wellbeing from April 2020 to April 2021 while controlling for the study covariates. PROCESS Model 1 allows testing the moderating effect with bootstrap confidence intervals. Race was also tested as a moderator for the relationship between one's fear of a COVID-19 infection and overall wellbeing. It was expected that psychosocial factors related to race and the pandemic would be strongly associated among Asian Americans, compared with Latinx Americans.

### Study 1 results

**Figure 1:**
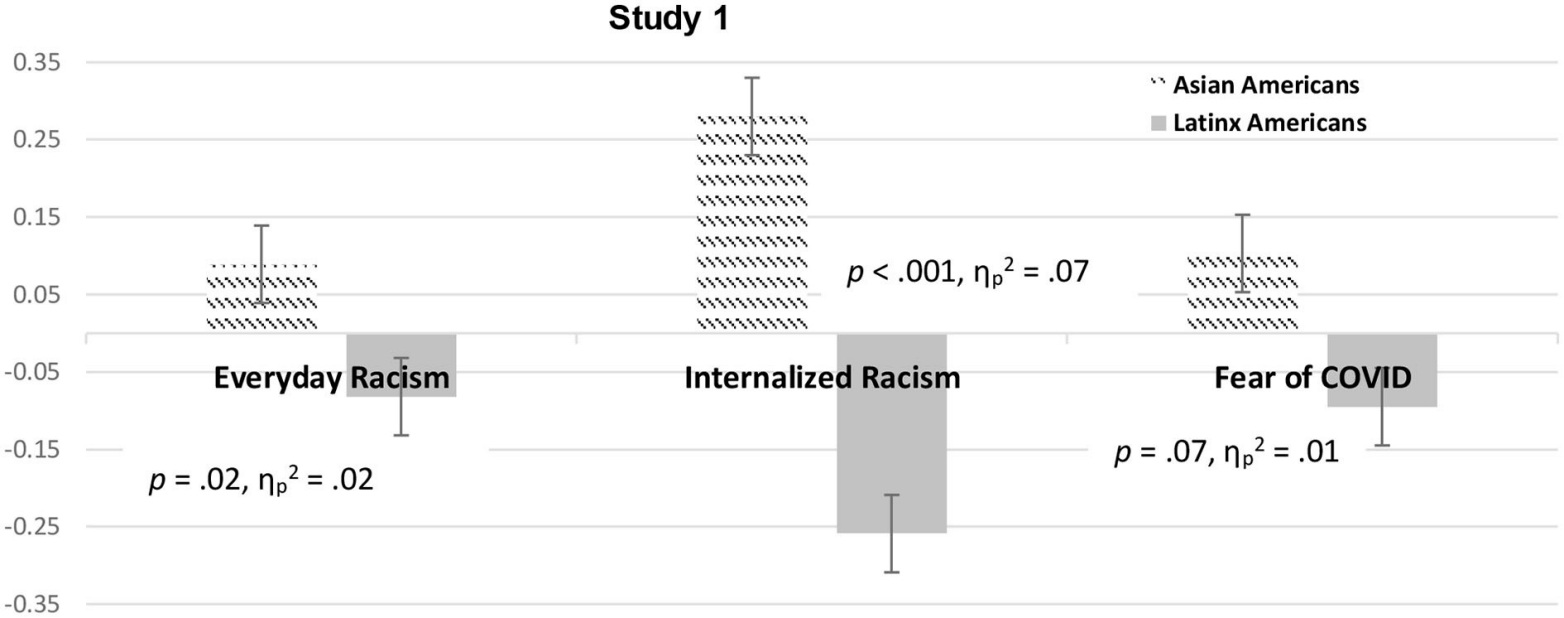
Psychosocial factor scores for Asian and Latinx Americans across study 1 controlling for covariates. *Note*. Psychosocial factor scores of Asian and Latinx Americans are shown for everyday racism, internalized racism, and fear of coronavirus (error bars show standard errors) controlling for everyday racism before the pandemic, gender, generation status, and socioeconomic status.

ANCOVA suggested that Asian and Latinx Americans were not significantly different in their levels of wellbeing when controlling for everyday racism before the pandemic and the other covariates. Asian Americans, however, did report higher levels of internalized racism, *F*_(1, 365)_ = 27.25, *p* < 0.001, $\eta_{p}^{2}$ = 0.07, and more everyday racism during the pandemic than Latinx Americans, *F*_(1, 365)_ = 6.84, *p* = 0.02, $\eta_{p}^{2}$ = 0.02. Asian Americans also showed more fear of the coronavirus, which trended toward significance, *p* = 0.07. Figure 1 shows the differences for everyday racism, internalized racism, and fear of the coronavirus between Asian and Latinx Americans.

The three moderating analyses assessed the role of race on the relationship between psychosocial factors (everyday racism, internalized racism, and fear of the coronavirus) and an individual's overall wellbeing. The results showed that race (Asian vs. Latinx Americans) was a significant moderator for the effects of everyday racism and fear of the coronavirus but not internalized racism. Notably, more everyday racism and heightened fear levels of the coronavirus negatively impacted the wellbeing of Asian Americans, but not Latinx Americans. For the relationship between everyday racism and wellbeing, the main independent variable (everyday racism) and the moderator (race) accounted for a significant amount of variance in wellbeing, *R*^2^ = 0.10, *F*_(7, 358)_ = 5.96, *p* < 0.001. See Table 3 for more details.

**Table 3:** Moderation results for everyday racism and race (Asian vs. Latinx Americans) with well-being as the outcome and everyday racism before the pandemic, gender, SES, and generation status as covariates for study 1.

**Effect**	***t***	***F***	***p***	***LLCI***	***ULCI***
Constant	0.70		0.48	−14.75	31.189
**Covariates**
Gender			0.10	−0.58	6.72
SES	5.26***		0.00	2.28	5.04
Generation status	0.98		0.33	−1.17	3.51
Everyday racism before the pandemic	−0.62		0.54	−0.15	2.50
**Main effects**
Everyday racism	1.75		0.08	0.08	0.01
Race (Asian vs. Latinx Americans)	1.94		0.05	0.86	0.00
**Interaction**
Everyday racism X race		4.26*	0.04		
Asian Americans	−1.27		0.20	−1.08	0.23
Latinx Americans	1.04		0.30	−0.34	1.09
Model summary	*R^2^*	*F*	*p*	*df1*	*df2*
	0.10	5.96	0.00	7	358

Gender: 1 = woman, 2 = man. SES = socioeconomic status. SES was from 1 to 10, with higher scores representing higher perceived SES. Generation Status: 1 = 1st generation, 2 = 1.5 generation, 3 = 2nd generation, 4 = 3rd generation, 5 = 4th and higher generation. Race: 1 = Asian American, 0 = Latinx American.

^*^p < 0.05,

^**^p < 0.01,

^***^p < 0.001.

**Figure 2:**
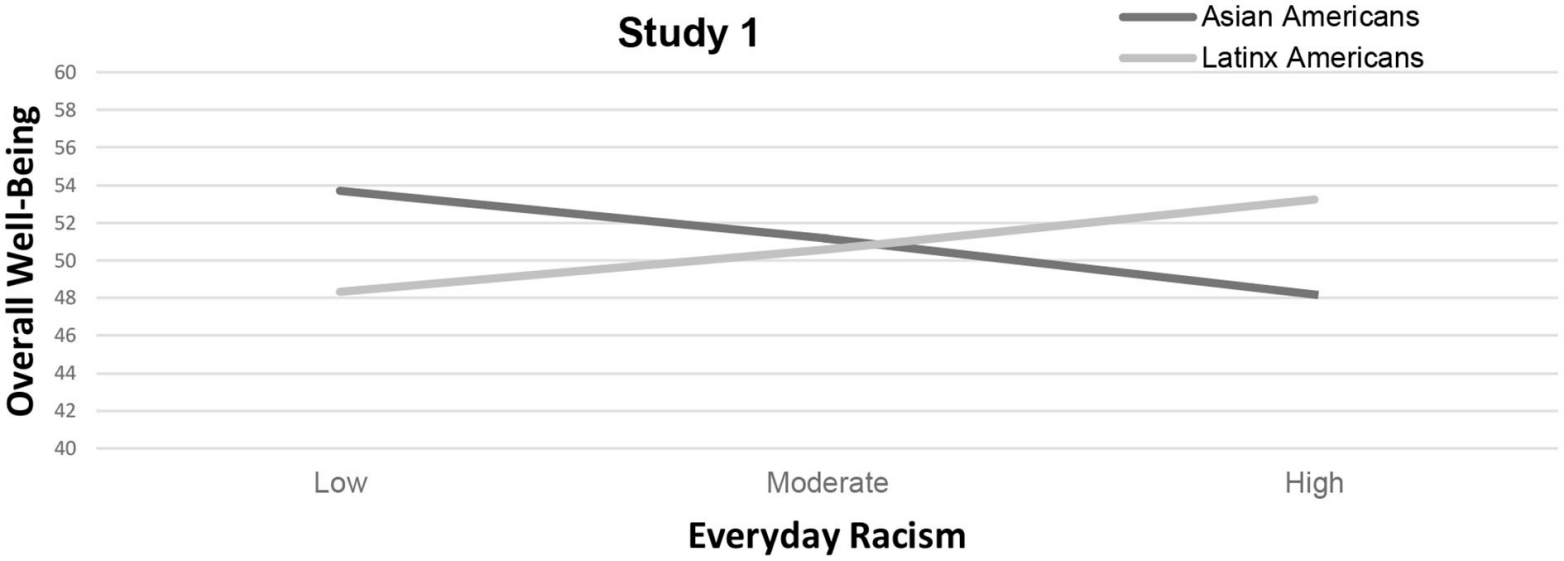
Changes in overall well-being as a function of race and everyday racism for study 1. *Note*. Results suggest race as a significant moderator (*p* < 0.05) for the relationship between everyday racism and overall well-being. Asian Americans experience lower overall well-being as a function of experiencing everyday racism compared to Latinx Americans.

To avoid potential multicollinearity with the interaction term, the variables were centered, and an interaction term between everyday racism and race was created (47). The results revealed a significant moderating effect of race, Δ*R*^2^ = 0.01, Δ*F*_(1, 358)_ = 4.26, *p* = 0.04, on the relationship between everyday racism and wellbeing, *b* = −0.80, *t* = −2.06, *p* = 0.04, 95% CI [−1.56, −0.04]. Furthermore, an examination of the simple slopes showed a crossover interaction with a negative relation for Asian Americans and a positive relation for Latinx Americans, that is, more experiences of everyday racism for Asian Americans showed lower wellbeing, whereas more experiences of everyday racism for Latinx Americans showed higher wellbeing. Figure 2 depicts the relationship between everyday racism and overall wellbeing as a function of race.

For the relationship between fear of the coronavirus and wellbeing, the main independent variable (fear of coronavirus) and the moderator (race) accounted for a significant amount of variance in wellbeing *R*^2^ = 0.12, *F*_(7, 358)_ = 7.21, *p* < 0.001. See Table 4 for more details. Again, the variables were centered, and an interaction term between fear of the coronavirus and race was created to avoid possible multicollinearity with the interaction term (47).

**Table 4:** Moderation results for fear of the coronavirus and race (Asian vs. Latinx Americans) with well-being as the outcome and everyday racism before the pandemic, gender, SES, and generation status as covariates for study 1.

**Effect**	***t***	***F***	***p***	***LLCI***	***ULCI***
Constant	4.75		0.00	32.35	78.02
**Covariates**
Gender	1.61		0.12	−0.66	6.66
SES	5.45***		0.00	2.28	5.04
Generation status	1.22		0.23	−1.17	3.51
Everyday racism before the pandemic	−0.80		0.43	−0.15	2.50
**Main effects**
Fear of the coronavirus	−3.29	−3.29**	0.00	0.08	0.01
Race (Asian vs. Latinx Americans)	1.94	−2.48*	0.01	0.86	0.00
**Interaction**
Fear of the coronavirus X race		7.33**	0.01		
Asian Americans	0.45		0.20	−1.08	0.23
Latinx Americans	−3.47***		0.00	−0.34	1.09
Model summary	*R^2^*	*F*	*p*	*df1*	*df2*
	0.12	7.21	0.00	7	358

Gender: 1 = woman, 2 = man. SES, socioeconomic status. SES was from 1 to 10, with higher scores representing higher perceived SES. Generation Status: 1 = 1st generation, 2 = 1.5 generation, 3 = 2nd generation, 4 = 3rd generation, 5 = 4th and higher generation. Race: 1 = Asian American, 0 = Latinx American.

^*^p < 0.05,

^**^p < 0.01,

^***^p < 0.001.

**Figure 3:**
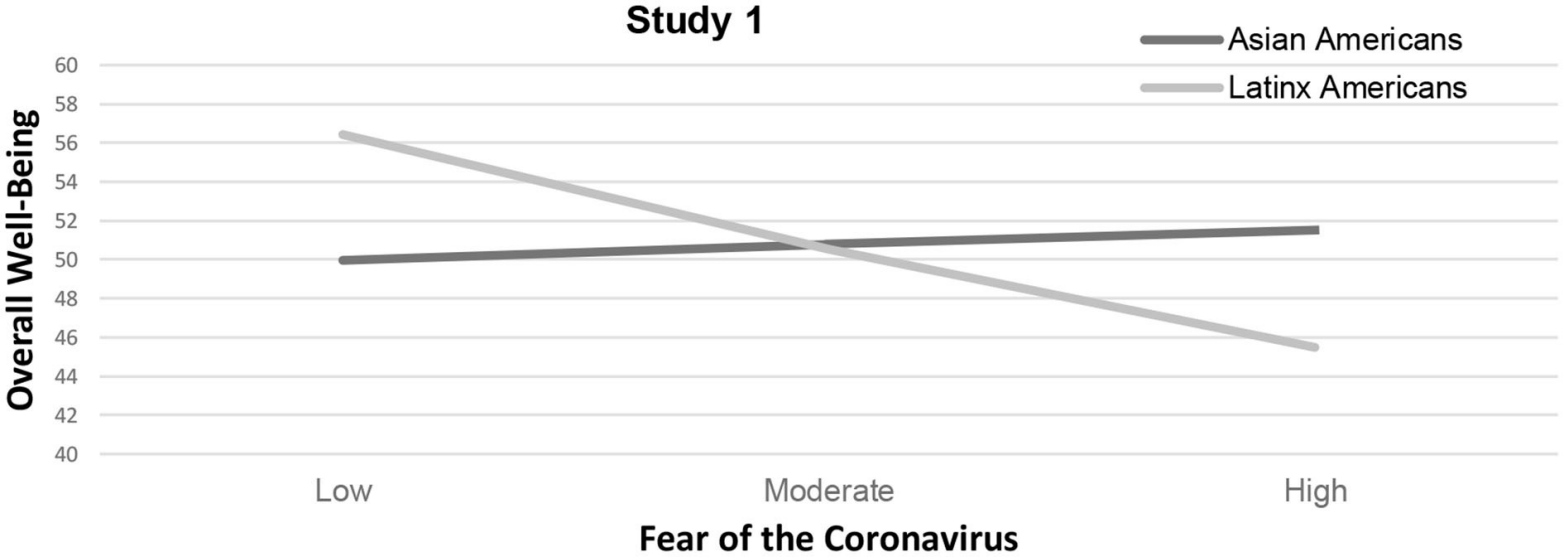
Changes in overall well-being as a function of race and fear of the coronavirus for study 1. *Note*. Results suggest race as a significant moderator (*p* < 0.05) for the relationship between fear of the coronavirus and overall well-being. Latinx Americans experienced lower overall well-being as a function of fear of the coronavirus compared to Asian Americans.

The results revealed a significant moderating effect of race, Δ*R*^2^ = 0.02, Δ*F*_(1, 358)_ = 7.33, *p* = 0.01, on the relationship between fear of the coronavirus and wellbeing, *b* = −0.80, *t* = −2.06, *p* = 0.04, 95% CI [−1.56, −0.04]. An examination of the slopes showed a significant effect for Latinx Americans, *b* = −0.84, *t* = −3.47, *p* = 0.001, 95% CI [−1.32, −0.36], but not for Asian Americans, that is, more severe fear of the coronavirus for Latinx Americans was associated with significantly lower overall wellbeing. Figure 3 shows the relationship between fear of the coronavirus and overall wellbeing as a function of race.

## Study 2

Study 2 (15 September 2021–29 March 2022) compared Asian and Latinx Americans during the months after the vaccine release and the lifting of major nationwide lockdown mandates. This study included the same measures as study 1, as well as additional assessments for distress over coronavirus victimization. Study 2 began data collection during a time when CDC studies provided further evidence that COVID-19 vaccines offered higher protection than a previous COVID-19 infection alone.

## Materials and methods

### Participants

Data for study 2 were collected online during the COVID-19 pandemic from the same university as in study 1. The final sample in this study consisted of 185 respondents between 18 and 50 years (*M*_age_ = 19.70, *SD* = 3.96), with a gender breakdown of 68.0% women, 31.1% men, and 0.9% “other.” The overall racial and ethnic breakdown of the sample was 47.0% Asian Americans and 53.0% Latinx Americans, which was similar to the distribution seen in study 1. Asian American participants reported significantly higher socioeconomic status than Latinx American participants. The sample included 41 foreign-born and 144 U.S.-born participants, and five participants were international students. All participants in the final sample resided in the United States during the pandemic and understood written English. A total of five participants were removed from the initial 158 participants for either being younger than 18 years or residing outside the United States. Table 5 lists the study demographic characteristics and descriptive statistics.

**Table 5:** Demographic characteristics and descriptives of study variables compared across asian and Latinx samples for study 2 (September 2021–April 2022).

**Variables**	**Race**					
	**Asian Americans**		**Latinx Americans**		**Full Sample**	
	***M***	***SD***	***M***	***SD***	***M***	***SD***
Age	19.74	2.11	19.90	3.35	19.74	3.74
SES	5.70_a_	1.52	5.13_b_	1.52	5.40	1.54
	*N*	%	*N*	%	*N*	%
**Gender**
Women	48	55.2	65	66.3	113	68.0
Men	33	37.9	33	33.7	66	31.1
Other	6_a_	6.9	0_b_	0.0	6	0.9
**Generation status**
1^st^	4	4.6	5	5.1	9	4.9
1.5	18_a_	20.7	9_b_	9.2	27	14.6
2^nd^	55	63.2	71	72.4	126	68.1
3^rd^	4	4.6	8	8.2	12	6.5
4^th^ or more	3	3.4	3	3.1	6	3.2
Other	3	3.4	2	2.0	5	2.7
Total	87	47.0	98	53.0	185	100

SES, socioeconomic status. SES was from 1 to 10, with higher scores representing higher perceived SES. Subscript letters denotes a subset of race categories whose column proportions or means differ significantly from each other at the 0.05 level.

#### Procedures

Participants were again recruited from the same undergraduate psychological participant pool and were compensated course credit for their participation in the study. Those who participated in study 1 were not eligible to participate in study 2. Procedures were similar to those in study 1 (see section 2.1.2 for details).

#### Measures

Refer to study 1 Measures section for information on everyday racism, internalized racism, and fear of the coronavirus scales.

##### Race and ethnicity

Specific racial and ethnic backgrounds were assessed as part of the demographic questionnaire, along with age, gender, SES, and generational status. Similar to study 1, these demographic questions were delivered at the end of the online questionnaire to avoid any possible priming effects. In addition, only monoracial people were included in this study since the main study variables were race-related.

##### Distress from coronavirus victimization

The Coronavirus Victimization Distress Scale (CVDS) (48) was used to assess levels of distress related to blaming and victimization of the pandemic. This measure was constructed after the launch of study 1 and, thus, was not included in the previous study. The CVDS consists of five items presenting scenarios describing possible victimization connected to the pandemic (e.g., “I have been teased or bullied because someone thought I was infected with the coronavirus”). The participants reported distress from these experiences ranging from 1 (*it never happened*) to 5 (*it happened and upset me quite a bit*). Fisher et al. (48) validated the CVDS using a diverse sample of Latinx and Asian American participants and showed a strong internal reliability of 0.91. Study 2 demonstrated comparable Cronbach's alpha values for Asian Americans (α = 0.85), Latinx Americans (α = 0.74), and the final sample (α = 0.81).

#### Statistical analyses

Partial correlation analysis along with analysis of variance (ANOVA) of test the demographic characteristics and study variables was used to examine differences among the sample while holding everyday experiences with racism before the pandemic constant (see Table 6). Similar to analyses of study 1, certain demographic characteristics were examined in relation to wellbeing and other moderating variables to determine any potential covariates for further analyses. Study 1 and study 2 identified the same three covariates—gender, generational status, and SES—to control for in the main statistical analyses.

**Table 6:** Correlations and ANOVA results for demographics and study variables for study 2 controlling for everyday racism before the pandemic.

**Variable**	**Everyday racism**	**Internalized racism**	**COVID fear**	**Victimization distress**	**Well-being**
		**Partial correlations** ***r***			
Age	0.05	−0.04	0.14	−0.05	0.03
SES	−0.04	−0.00	0.06	−0.05	0.28***
Generation status	0.06	0.02	−0.10	−0.12	−0.02
		**ANOVA** ***F***			
Gender	1.92	1.75	11.27${}_{\text{w}}^{**}$	0.30	6.02${}_{\text{m}}^{*}$
Race	0.22	1.24	5.15${}_{\text{a}}^{*}$	3.41	1.61

SES, socioeconomic status. SES was from 1 to 10, with higher scores representing higher perceived SES. Generation Status: 1 = 1st generation, 2 = 1.5 generation, 3 = 2nd generation, 4 = 3rd generation, 5 = 4th and higher generation. Gender: subscript m denotes higher mean for men, subscript w denotes higher mean for women. Race: subscript a denotes higher mean for Asian Americans.

^*^p < 0.05,

^**^p < 0.01,

^***^p < 0.001.

ANCOVAs were conducted on the same main outcome and moderating variables as in study 1: wellbeing, everyday racism, internalized racism, and fear of the coronavirus. Furthermore, we examined distress related to COVID-19 victimization, using the same covariates as in study 1. We used SPSS PROCESS Macro (46) to test whether race moderated the associations between everyday racism, internalized racism, fear of the coronavirus, and distress victimization, and an individual's overall wellbeing.

### Study 2 results

The comparison analyses show that Asian and Latinx Americans varied across gender, generational status, and SES, but not age (see Table 2). Considering gender, both Asian and Latinx American samples had more women than men. Regarding SES, Asian Americans still reported substantially higher status than Latinx Americans, although the differences were less than those in study 1. Considering generational status, the Asian American sample had approximately 11% more people reporting a 1.5 status compared with the Latinx American sample. See Table 2 for more detailed demographic characteristics.

**Figure 4:**
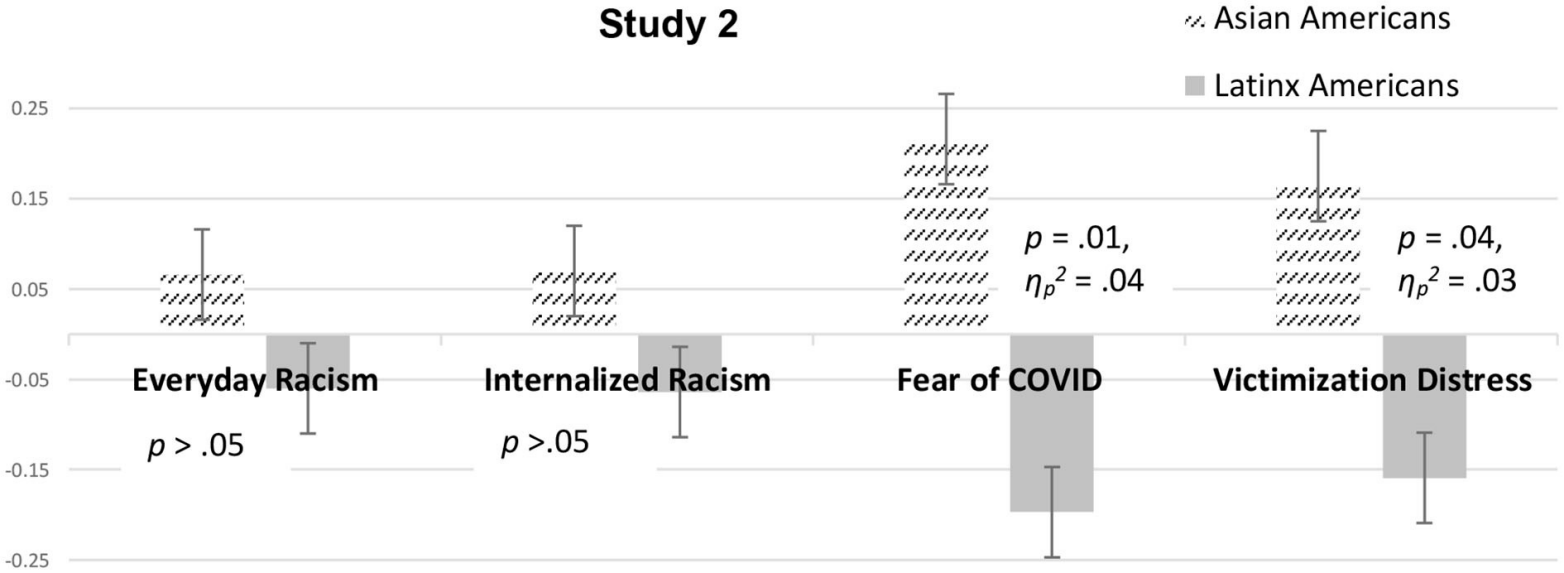
Psychosocial factor scores for Asian and Latinx Americans across study 2 controlling for covariates. *Note*. Psychosocial factor scores of Asian and Latinx Americans are shown for everyday racism, internalized racism, fear of coronavirus, and victimization distress (error bars show standard errors), controlling for everyday racism before the pandemic, gender, generation status, and socioeconomic status.

Similar to study 1, comparison ANCOVAs indicated Asian and Latinx Americans did not differ significantly on wellbeing. Unlike study 1, there were no racial differences with internalized racism and everyday racism, when controlling for the mentioned covariates. Nevertheless, Asian participants did report more fear of the coronavirus, *F*_(1, 152)_ = 6.31, *p* = 0.01, $\eta_{p}^{2}$ = 0.04, and distress from coronavirus victimization, *F*_(1, 152)_ = 4.14, *p* = 0.04, $\eta_{p}^{2}$ = 0.03, than Latinx participants, that is, Asian Americans were comparatively more distressed about overt discrimination related to COVID-19. Figure 4 depicts the differences between everyday racism, internalized racism, fear of the coronavirus, and victimization distress reported by Asian and those reported by Latinx Americans.

Moderating analyses suggested that race did not significantly moderate the relationships among everyday racism, internalized racism, fear of coronavirus, and wellbeing. Despite that result, race moderated the effect of distress from coronavirus victimization on wellbeing, *F*_(1, 184)_ = 6.93, *p* = 0.01, $\eta_{p}^{2}$ = 0.04. For the relationship between coronavirus victimization distress and wellbeing, the main independent variable (coronavirus victimization distress) and the moderator (race) accounted for a significant amount of wellbeing variance, *R*^2^ = 0.16, *F*_(7, 145)_ = 3.86, *p* < 0.001. See Table 7 for more details.

**Table 7:** Moderation results for victimization distress and race (Asian vs. Latinx Americans) with well-being as the outcome and everyday racism before the pandemic, gender, SES, and generation status as covariates for study 2.

**Effect**	***t***	***F***	***p***	***LLCI***	***ULCI***
Constant	4.08		0.48	−14.75	31.189
Covariates					
Gender	0.31		0.76	−0.58	6.72
SES	3.24**		0.00	2.28	5.04
Generation status	−0.56		0.58	−1.17	3.51
Everyday racism before the pandemic	−0.58		0.56	−0.15	2.50
Main effects					
Victimization distress	−2.72		0.01	0.08	0.01
Race (Asian vs. Latinx Americans)	−2.47		0.01	0.86	0.00
Interaction					
Victimization distress X race		5.35*	0.02		
Asian Americans	−0.71		0.20	−2.19	1.03
Latinx Americans	−3.00**		0.00	−7.46	−1.53
Model summary	*R^2^*	*F*	*p*	*df1*	*df2*
	0.16	3.86	0.00	7	145

Gender: 1 = woman, 2 = man. SES = socioeconomic status. SES was from 1–10, with higher scores representing higher perceived SES. Generation Status: 1 = 1st generation, 2 = 1.5 generation, 3 = 2nd generation, 4 = 3rd generation, 5 = 4th and higher generation. Race: 1 = Asian American, 0 = Latinx American.

^*^p < 0.05,

^**^p < 0.01,

^***^p < 0.001.

**Figure 5:**
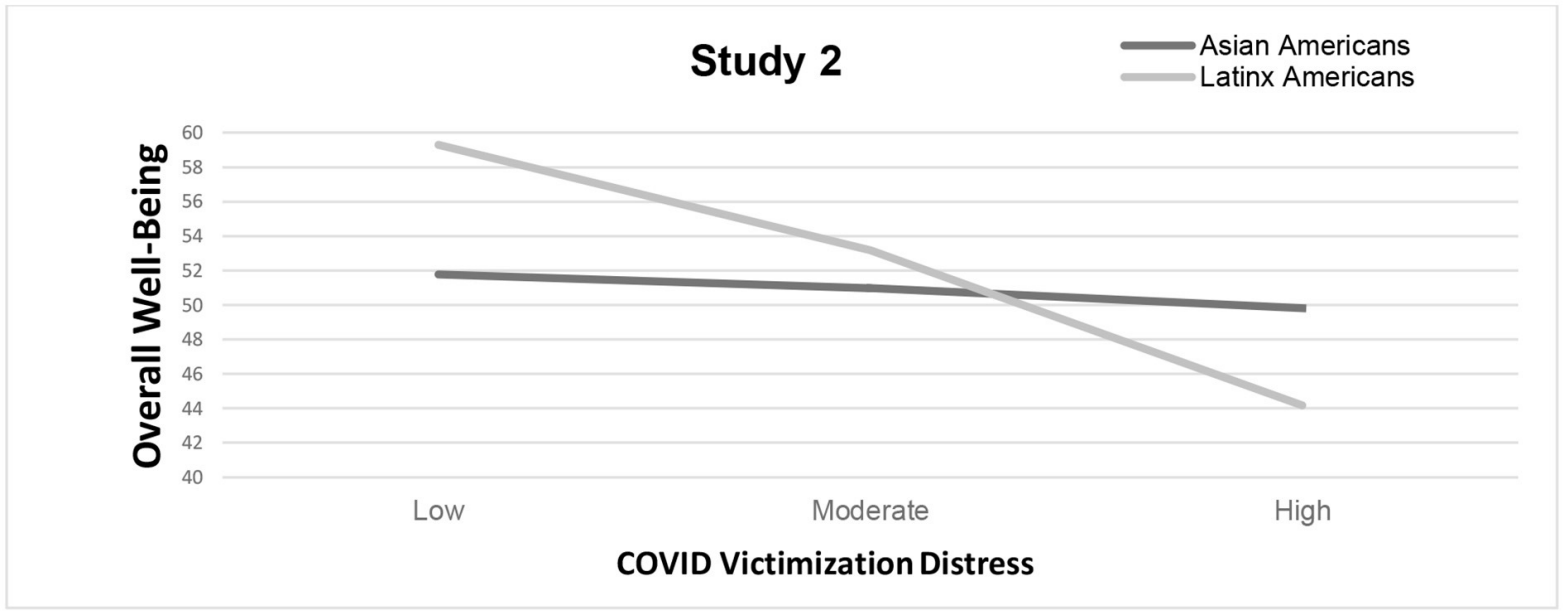
Changes in overall well-being as a function of race and everyday racism for study 2 (September 2021–April 2022). *Note*. Results suggest race as a significant moderator (*p* < 0.05) for the relationship between COVID-19 victimization distress and overall well-being. Latinx Americans experienced lower overall well-being as a function of COVID-19 victimization distress compared to Asian Americans.

To avoid potential multicollinearity with the interaction term, all primary variables were centered, and an interaction term between the distress of coronavirus victimization and race was created (41). The results revealed a significant moderating effect of race, Δ*R*^2^ = 0.03, Δ*F*_(1, 145)_ = 5.35, *p* = 0.02, on the relationship between coronavirus victimization distress and wellbeing, *b* = 1.96, *t* = 2.31, *p* = 0.02, 95% CI [0.28, 3.63]. A closer examination of the simple slopes showed that higher levels of distress negatively correlated with wellbeing among Latinx Americans, *t* = −3.00, *p* = 0.003, 95% CI [−7.46, −1.53], but Asian Americans did not share those effects. This finding is slightly contradictory to our predictions, given that the relationship was stronger and more significant for Latinx Americans than for Asian Americans. Figure 5 shows the relationship between victimization distress and overall wellbeing as a function of race.

### Overall discussion and implications

Undoubtedly, the COVID-19 pandemic—from the beginning to the end—was a psychologically distressing time for many people. To this point, findings from both studies demonstrate that Asian and Latinx Americans experienced reduced levels of wellbeing during the recent pandemic; however, no significant racial differences in wellbeing were observed between the groups. Nevertheless, it is worth noting that nearly half of the Asian and Latinx Americans from the samples reported reduced levels of wellbeing in general, with almost one in six meeting the cutoff for clinical depression in study 1 and almost one in 10 in study 2. Despite the similarities between Asian and Latinx Americans, their reduced levels of wellbeing, which suggested probable depression in many cases, were nearly two times higher than the rates seen in the general population before the pandemic.

This sudden drop in wellbeing and rise in depressive rates warrant further attention from U.S. officials since it seems to indicate a growing public health concern for Asian Americans, as well as Latinx Americans. Another point worth noting is that for Asian Americans, rates of reduced wellbeing were climbing between the time frames of the studies (43.8–49%), whereas for Latinx Americans, rates of reduced wellbeing were lowering (52.6–40.8%). This considerable reduction in wellbeing, especially if left unaddressed, might increase Asian Americans' susceptibility to psychological disorders and problematic behaviors, such as depression and addictions. In turn, Asian Americans might seek out healthcare services at a greater frequency and at much higher rates than before the pandemic. Barriers to cultural competent services (e.g., language assistance programs) may need additional improvements in regard to assisting Asian Americans in dealing with the aftermath of the pandemic.

Furthermore, the fast and attention-grabbing violence toward Asian Americans that was widely publicized during the pandemic, corroborating the findings of study 1 that indicated a significant upward shift in everyday racism for Asian Americans as compared with Latinx Americans. Asian Americans also reported relatively higher levels of internalized racism throughout the duration of the studies. The exponential uptick of hate crimes, or fast violence, against Asian Americans during the pandemic perhaps spilled over into the racism-related slow violence reported by the participants in both the studies, based on their everyday exchanges, internalized hate, and/or victimization distress. In addition, moderating analyses showed that an individual's race was a significant moderator for the relationship between everyday racism and overall wellbeing. Not only was there an upward shift in experiences of everyday racism but also this effect seemed more negatively linked to Asian Americans' levels of wellbeing. This general trend in both the studies highlights the unique burden that racism places on many people of color. Latinx Americans may have not experienced more distress from COVID-19-related victimization, but Latinx Americans did report more distress and lower wellbeing than Asian Americans.

In response the racial inequities during the pandemic, the APA issued a call for an increase in training, awareness, research, and the creation of clinical tools that are culturally adapted for people of color (49, 50). One way the APA Task Force on Race and Ethnicity (49) suggested that psychologists should manage these sensitive issues through culture-specific treatments and other indigenous healing approaches (e.g., healing circles). Researchers premise the use of healing circles around the need to provide safer spaces for people of color to learn positive coping skills and process stress-related experiences of racism. Limited research suggests that those individuals who take part in healing circles may feel more confident and have more skills to cope with future stressful experiences of racism, especially in connection to racism (51). Other traditional approaches for treating people of color can also include culturally adapted group psychotherapy sessions that integrate specific recognition and processing of racism-related experiences, such as microaggressions (52). Researchers have found that psychotherapy that centers on a multiculturally competent framework improves the working alliance and clinical outcomes of Asian and Latinx Americans. Past scholarly literature on this topic suggests that integrating client-defined group membership and intersecting identities are critically important for therapeutic outcomes (53, 54). These findings would suggest that during a time in which Asian Americans are suffering due to increased levels of experienced and internalized racism, coronavirus distress, and victimization, integrating experiences of racism through a multicultural competent lens into group psychotherapy or healing circles may prove to be an invaluable resource for the Asian American community to heal from the devastating effects of the pandemic-associated racism.

In comparison to Latinx Americans, Asian Americans reported more worry about the coronavirus throughout the pandemic, particularly in relation to the fear of infection. This increase in generalized fear and anxiety, along with navigating the blindsiding shift in racism, plausibly made many Asian Americans more susceptible to coronavirus infections and other ailments. In 2020, one in seven Asian American deaths were related to COVID-19, and Asian Americans were overrepresented in mortality rates compared with non-Hispanic whites (55). Emerging statistics show that Asian Americans who were hospitalized also presented with more severe infections and were more likely to die from the coronavirus than non-Hispanic whites. Interestingly, moderation results from study 1 indicated that one's fear severity had more of a negative effect on the overall wellbeing of Latinx Americans, but not Asian Americans, that is, Latinx Americans who had a more severe fear of the coronavirus had significantly lower wellbeing. Although this finding us slightly contrary to our predictions, the overall results suggest fear and anxiety of the coronavirus are important when considering people of color's wellbeing.

Differences in racism-related factors were not significant when the COVID-19 vaccines were widely available during study 2. As predicted, Asian Americans reported more distress from coronavirus victimization than Latinx Americans. Much of the distress Asian Americans experienced appeared to be more related to being blamed for the coronavirus outbreak. Overall, though, these results suggest that the psychological impact of COVID-19 might be, by comparison, more robust for Asian Americans; however, Latinx Americans who experience more fear and blame for the coronavirus experienced lower levels of wellbeing. During the later months of the pandemic, major efforts were launched to stop hate against Asian American communities in the United States (i.e., #StopAAPIHate and Biden's Hate Crimes Bill). Perhaps these public gestures to stop racism against Asian Americans in the United States also mitigated some of the everyday and internalized racism effects, whereas Latinx Americans did not experience the same large-scale efforts during the pandemic.

## Limitations and conclusions

We examined the factors related to psychosocial experiences with racism among Asian Americans and the coronavirus during the critical months of the pandemic as compared with Latinx Americans. The results from these studies should be interpreted considering research limitations. First, the data were collected as online questionnaires from two college samples at the same university. Therefore, generalizability interpretations of our findings might be limited to an emerging adulthood experience, particularly young people completing an undergraduate degree. Future studies should assess other demographic groups since many effects of pandemic may continue to linger throughout the United States. In addition, our studies were cross-sectional designs examining separate samples at different time points of the pandemic. These studies only considered associations between psychosocial factors and wellbeing, so future longitudinal methodologies will help clarify the directionality of these effects.

In sum, the takeaway message from the current findings is that Asian Americans have experienced and still are experiencing multi-faceted forms of racism. During the beginning months of the pandemic, Asian Americans in our samples reported significantly more experiences with the slow violence of racism than Latinx Americans; and while some people may shutter at the very thought of calling words a form of violence, this term is meant to capture an important truth: hateful words *can* be weapons—ones that often inflict emotional harm and pain far beyond what the human eye is able to observe. In our samples, we were able to glimpse some of these negative effects that racist and xenophobic words and acts can have on Asian American people, many of whom experienced more consistent fear and distress during the time of the pandemic. Lately, it seems most instances of fast violence against Asian Americans might have, for a time, been mitigated, but the unseen effects of racism will likely endure for many people of color.

## Data availability statement

The raw data supporting the conclusions of this article will be made available by the authors, without undue reservation.

## Ethics statement

The studies involving human participants were reviewed and approved by Institutional Review Board. The patients/participants provided their written informed consent to participate in this study.

## Author contributions

GW-P was lead author and contributed to the conception, design, organized the database, performed statistical analyses, and wrote the draft of the manuscript. AB contributed to the conception and design of the study, and organized the database. AK and MS wrote sections of the manuscript. All authors contributed to the manuscript revision, read, and approved the submitted version.

## Funding

The publication fees for this article were supported by the UNLV University Libraries Open Article Fund.

## Conflict of interest

The authors declare that the research was conducted in the absence of any commercial or financial relationships that could be construed as a potential conflict of interest.

## Publisher's note

All claims expressed in this article are solely those of the authors and do not necessarily represent those of their affiliated organizations, or those of the publisher, the editors and the reviewers. Any product that may be evaluated in this article, or claim that may be made by its manufacturer, is not guaranteed or endorsed by the publisher.
